# Perturbation of cellular proteostasis networks identifies pathways that modulate precursor and intermediate but not mature levels of frataxin

**DOI:** 10.1038/srep18251

**Published:** 2015-12-16

**Authors:** Joseph F. Nabhan, Renea L. Gooch, Eugene L. Piatnitski Chekler, Betsy Pierce, Christine E. Bulawa

**Affiliations:** 1 Rare Disease Research Unit, Worldwide Research and Development, Pfizer, 610 Main Street, Cambridge, MA 02139, USA; 2 Worldwide Medicinal Chemistry, Pfizer, 610 Main Street, Cambridge, MA 02139, USA; 3 Worldwide Medicinal Chemistry, Pfizer, Eastern Point Road, Groton, CT 06340, USA

## Abstract

Friedreich’s Ataxia is a genetic disease caused by expansion of an intronic trinucleotide repeat in the frataxin (*FXN*) gene yielding diminished FXN expression and consequently disease. Since increasing FXN protein levels is desirable to ameliorate pathology, we explored the role of major cellular proteostasis pathways and mitochondrial proteases in FXN processing and turnover. We targeted p97/VCP, the ubiquitin proteasome pathway (UPP), and autophagy with chemical inhibitors in cell lines and patient-derived cells. p97 inhibition by DBeQ increased precursor FXN levels, while UPP and autophagic flux modulators had variable effects predominantly on intermediate FXN. Our data suggest that these pathways cannot be modulated to influence mature functional FXN levels. We also targeted known mitochondrial proteases by RNA interference and discovered a novel protease PITRM1 that regulates intermediate FXN levels. Treatment with the aforementioned chemical and genetic modulators did not have a differential effect in patient cells containing lower amounts of FXN. Interestingly, a number of treatments caused a change in total amount of FXN protein, without an effect on mature FXN. Our results imply that regulation of FXN protein levels is complex and that total amounts can be modulated chemically and genetically without altering the absolute amount of mature FXN protein.

In the genetic disease Friedreich’s Ataxia (FRDA), a homozygous GAA (Guanine-Adenine-Adenine) trinucleotide expansion in intron 1 of the *FXN* nuclear locus partly silences *de novo* transcription leading to a reduction in intracellular levels of FXN protein^1^,^2^. The *FXN* transcript is translated into a cytosolic precursor protein (pFXN) that is rapidly imported into mitochondria, where it is further processed from an intermediate form (iFXN) to a mature protein (mFXN), resident in the mitochondrial matrix^3^. mFXN plays a role in activation of a desulfurase complex to generate iron-sulfur (Fe-S) clusters^4^, which serve as prosthetic groups that functionally enable a large number of mitochondrial and extra-mitochondrial proteins^5^. Low levels of mFXN reduce Fe-S cluster synthesis, ultimately causing pathology in affected tissues^6^. Increasing levels of mFXN is therefore desirable, and is likely to be therapeutic for FRDA patients.

Most approaches to date have focused on potentiating expression of FXN from the pathogenic *FXN* locus. In contrast, the possibility of post-translational modulation of FXN levels has not been sufficiently explored. Two previous reports suggested that the ubiquitin proteasome pathway (UPP) pathway degrades pFXN^7^,^8^, and that UPP inhibition can spare pFXN from degradation to ultimately increase mFXN levels. Herein, we extend the study of proteostasis pathways on FXN processing and degradation. Using multiple cell lines and FRDA patient-derived cells, we examined the effect of chemical inhibitors of the UPP and other major nodes in the proteostasis network, including key regulators of autophagy and p97/VCP (valosin-containing protein), on endogenous mFXN protein levels. While UPP inhibition did not increase levels of FXN, some treatments augmented total FXN levels through upregulation of pFXN and/or iFXN, suggesting complex modulation of FXN import and processing in mitochondria. Uncoupling of mitochondrial membrane potential and suspected alteration of mitochondrial pH, both of which are known to impact mitochondrial import^9^,^10^ and processing^11^, reproduced some of the phenotypes elicited by proteostasis modulators. We further carried out an siRNA screen targeting known mitochondrial proteases and discovered that knockdown of PITRM1 augmented total FXN, again by increasing iFXN. Although we do not dissect the detailed molecular mechanisms that regulate FXN processing in this current study, our data highlights the important finding that mFXN level is recalcitrant to change whereas precursor levels fluctuate. Thus, measurement of total FXN does not predict mFXN level, underscoring the need to characterize potential FXN enrichment therapies using methods that monitor FXN processing.

## Results

### The mitochondrial protein maturation machinery does not limit mFXN accumulation

FXN is expressed in the cytoplasm as a 210 amino acid (AA) precursor protein (pFXN; 23 KDa) that is translocated into mitochondria where it is processed by two consecutive steps into iFXN (FXN 42–210; 19 KDa) and finally mFXN (81–210; 14.2 KDa), which is functional^12^,^13^. Post-translational regulation of mFXN levels remains elusive, but the half-life of mFXN is long^14^, suggesting that degradation of mFXN is not a major control point. The mechanism of turnover of pFXN and iFXN has not been studied but corresponding half-lives, as they relate to maturation of FXN, were previously estimated to be ~10 min and 2 h, respectively^14^. Our aim was to explore the possibility that the levels of pFXN and/or iFXN are regulated by degradation; if so, modulation of these pathways could ultimately increase mFXN.

**Figure 1:**
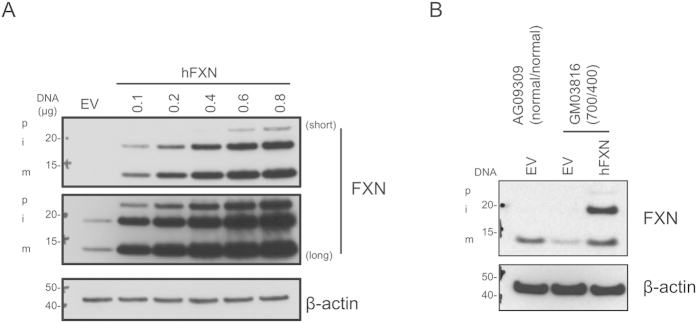
The FXN maturation machinery is not limiting in healthy and FRDA cells. (**A**) 293T cells were seeded in 6-well plates and transfected with the indicated amounts of hFXN expression construct, and supplemented with empty vector (EV) to 1 μg total per well. Cell lysates were prepared 36 h later and analyzed by immunoblotting with antibodies to the indicated proteins. Multiple exposures are shown (short and long) to illustrate accumulation of different forms of FXN. (**B**) Healthy donor human fibroblasts (AG09309) or FRDA patient-derived cells (GM03816) were transfected with 1 μg of EV or hFXN expression construct, as indicated. Lysates were prepared 36 h later and analyzed as in panel A. p, i, and m indicate precursor, intermediate, and mature forms, respectively. The numbers of GAA expansions in intron 1 of *FXN* (allele 1/allele 2) are noted. All gels were run under the same experimental conditions, as outlined in the Materials and Methods section.

We first eliminated the possibility that the FXN maturation machinery may limit steady state levels of mFXN. 293T cells were transfected with increasing amounts of a construct expressing full length human FXN (hFXN). Despite expression of over 100-fold FXN, at the highest transfected amount of hFXN, mitochondria appeared capable of processing at least 50% of the total protein into the mature form, suggesting that the processing machinery is not limiting and that it can mediate maturation of excess FXN protein (Fig. 1A). In comparison with empty vector (EV) -transfected cells, a large amount of mFXN was present in the hFXN-transfected cells. To further confirm that exogenous FXN protein can be processed into mature form in a FRDA disease background, we transfected FRDA fibroblasts (GM03816) with hFXN or EV. FRDA cells display partial silencing of the *FXN* locus due to the presence of an intronic expansion, thereby leading to >70% suppression in levels of FXN. FRDA and control fibroblasts transfected with construct alone (EV) displayed a pronounced difference in FXN levels, as expected (Fig. 1B; EV transfected lanes). Importantly, a clear increase was observed in FRDA fibroblasts transfected with hFXN -expressing construct that raised mFXN to levels higher than those in control fibroblasts from healthy donors, further confirming that the maturation machinery can process more than endogenous levels of FXN in an FRDA disease background (Fig. 1B).

### Inhibition of human p97/VCP/cdc48 by DbeQ or uncoupling of mitochondrial membrane potential leads to accumulation of pFXN but no change in mFXN levels

**Figure 2:**
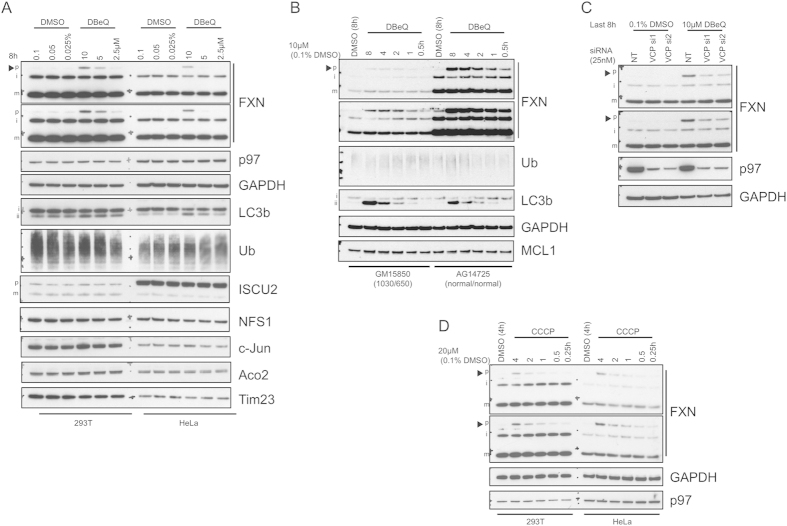
p97 inhibition with DBeQ or CCCP treatment causes an increase in pFXN but mFXN levels remain unaltered. (**A**) 293T or HeLa cells were treated with increasing amounts of DBeQ or vehicle (DMSO) for 8 h. Cell lysates were harvested and analyzed by immunoblotting with the indicated antibodies. (**B**) FRDA patient-derived lymphoblasts (GM15850) or healthy donor lymphoblasts (AG14725) were treated with 10 μM DBeQ for the indicated time periods or DMSO for 8 h. Lysates were then analyzed by immunoblotting. (**C**) 293T cells were transfected with 25 nM non-targeting (NT) or p97/VCP-targeting siRNA 1 or 2 for 64 h before treatment with 10 μM DBeQ or DMSO vehicle for 8 h and analysis of lysates by western blotting with the indicated antibodies. (**D**) 293T or HeLa cells were subjected to a time course treatment with 20 μM CCCP as indicated or with DMSO for 4 h and later analyzed by immunoblotting. Multiple exposures are shown to illustrate non-saturating levels of different forms of FXN protein in different cell types. Arrowheads indicate pFXN immunopositive bands. All gels were run under the same experimental conditions, as outlined in the Materials and Methods section.

p97/VCP/cdc48 (p97) plays key roles in proteostasis such as integration of signals leading to UPP or autophagy–mediated degradation and modulation of the stability of proteins associated with the outer mitochondrial membrane (OMM)^15^,^16^. We treated 293T and HeLa cells with a well characterized p97 inhibitor, DBeQ (N2,N4-Bis(phenylmethyl)-2,4-quinazolinediamine)^17^,^18^ and examined the effect on FXN accumulation. Treatment for 8 h led to robust accumulation of pFXN compared to DMSO -treated controls, but no increase in mFXN was observed (Fig. 2A). In the mitochondria, FXN interacts with components of the Fe-S biogenesis machinery, all of which are encoded by nuclear genes^19^. DBeQ did not alter levels or processing of known mitochondrial interaction partners of FXN, ISCU2 and NFS1, as well as mitochondrial aconitase (Aco2), a protein whose activity relies on FXN functionality (Fig. 2A). Additionally, no effect was detected on total poly-ubiquitinated (pUb) protein levels or the UPP substrate c-Jun. Lipidated LC3b (LC3b-II), which displays increased electrophoretic mobility, accumulated, indicative of blockade of autophagic flux, consistent with previous observations^18^. We then compared treatment with DBeQ of healthy and FRDA patient-derived lymphoblasts at multiple time points. Analysis of corresponding cell lysates showed that the time course and extent of pFXN accumulation was disease-independent (Fig. 2B). An overall increase in amount of FXN protein in cells treated with p97 inhibitor was observed, suggesting the presence of degradation pathways that control pFXN and/or iFXN levels. A time-dependent increase in LC3b-II was detected, consistent with the effect of DBeQ in 293T and HeLa cells.

To demonstrate that the effect of DBeQ is mediated by p97, we performed siRNA transfections in 293T cells targeting p97. As shown in Fig. 2C (lanes 1–3), knockdown of p97 did not reproduce the effect seen using DBeQ, raising the possibility that the effect on FXN is due to modulation of a different target. To test this possibility, we treated cells subjected to p97 knockdown with DBeQ (Fig. 2C, last 3 lanes). The increase in pFXN is clearly dependent on p97 and is significantly reduced in cells transfected with two different oligos targeting p97. One possible explanation is that DBeQ locks p97 in an inhibitory state that sequesters proteins essential for FXN import into the mitochondria, ultimately yielding an increase in amounts of accumulated pFXN, or that p97 is directly involved in facilitating import of pFXN, and that binding to DBeQ impedes its ability to release FXN. p97 is generally regarded as a hub that mediates interaction with multiple protein complexes depending on cellular localization^15^,^16^. It is therefore not surprising that p97 inhibitors can yield distinct effects on p97-mediated cellular processes^20^ that cannot be reproduced with p97 knockdown.

Because p97 may regulate mitochondrial protein import^21^,^22^, we asked whether perturbation of mitochondrial membrane potential, which was previously shown to influence mitochondrial protein processing and turnover^10^, would yield a similar effect. Indeed, treatment with the protonophore CCCP (carbonyl cyanide m-chlorophenyl hydrazine), caused rapid accumulation of pFXN without any detectable change in the level of p97 (Fig. 2D).

### Inhibition of the UPP causes reduction in intermediate FXN without altering mature FXN levels

**Figure 3:**
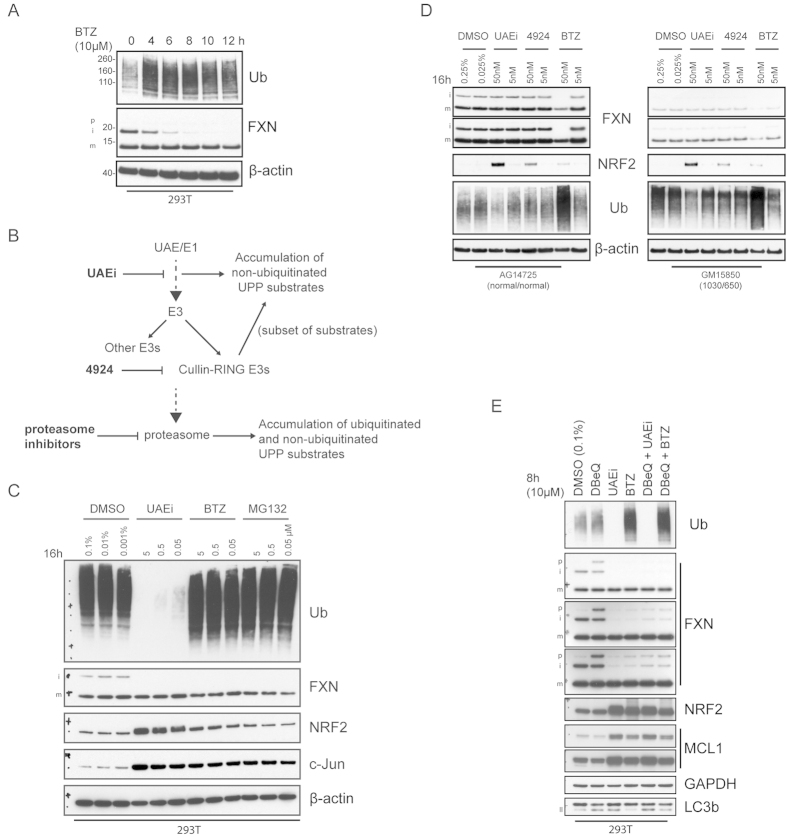
Inhibition of the ubiquitin proteasome pathway does not increase mFXN protein in 293T cells or in FRDA patient-derived lymphoblasts. (**A**) 293T cells were treated with 10 μM BTZ for the indicated times and then subjected to western analysis. (**B**) Schematic representation of the UPP and the expected effect of node inhibitors on substrate levels. Inhibitors noted in bold letters. (**C**) Dose-response western analysis of 293T cells treated with vehicle (DMSO), UAE inhibitor (UAEi), or the proteasomal inhibitors BTZ or MG132 for 16 h. (**D**) FRDA patient-derived (GM15850) or healthy match (AG14725) lymphoblasts were treated for 16 h with UAEi, 4924, or BTZ and subjected to analysis with the indicated antibodies. The numbers of GAA expansions in intron 1 of the *FXN* gene (allele 1/allele 2) are noted. (**E**) 293T cells were treated with inhibitors or combinations of inhibitors as indicated for 8 h. Lysates were harvested and analyzed by immunoblotting. Multiple exposures are shown to illustrate non-saturating levels of different forms of FXN protein in different cell types. All gels were run under the same experimental conditions, as outlined in the Materials and Methods section.

We next investigated the role of the UPP in endogenous FXN degradation. Treatment of 293T cells for 24 h with proteasomal inhibitors Bortezomib (BTZ) and MG132 did not alter the levels of mFXN (Fig. S3A). A very small increase in pFXN was detected, but only when a large amount of lysate was analyzed, causing saturation of the mFXN signal. Interestingly, iFXN was robustly diminished. Time course analysis showed that iFXN levels rapidly declined and were barely detectable after 10 h of proteasomal inhibition (Fig. 3A). mFXN levels remained unaltered during the course of the treatment.

To further dissect the effect of UPP inhibition on all forms of FXN levels, we expanded our studies to include additional inhibitors that act upstream of the proteasome (Fig. 3B). In our analysis, we typically avoided exceeding 24 h of compound treatment due to the cytotoxic effects of UPP inhibition. Ubiquitin activating enzyme (UAE)/E1 inhibition blocks formation of the E1-ubiquitin thioester in the first step of the ubiquitination pathway^23^ leading to accumulation of substrates turned over by the proteasome (Fig. 3B). Treatment of 293T cells with the UAE inhibitor (UAEi) reduced iFXN, as observed for the proteasomal inhibitors MG132 and BTZ, and did not affect mature FXN levels (Fig. 3C). To confirm the activities of the UPP inhibitors, we monitored the levels of multiple known proteasomal substrates. As expected, levels of NRF2 and c-Jun were increased compared to DMSO–treated controls (Fig. 3C). Additionally, polyubiquitinated protein levels were strongly diminished or enhanced following UAEi or MG132/BTZ treatment, respectively.

We next explored whether the effects of UPP inhibition are altered in FRDA patient-derived cells. We also included in this analysis treatment with MLN4924 (4924) which inhibits a subset of E3 enzymes, the Cullin-RING ubiquitin ligases (Fig. 3B)^24^. We monitored levels of NRF2 in our analysis because corresponding turnover is mediated by a member of this family of ubiquitin ligases^25^. FXN levels were unaltered in response to treatment with UAEi or MLN4942. Surprisingly, proteasomal inhibition with BTZ had an adverse effect on both iFXN and mFXN levels, despite treatment with low concentrations of BTZ (5 nM and 50 nM). The reduction in mFXN is unexpected given the long half-life reported for mFXN^14^. However, it is noteworthy that in our experiments, lymphoblasts were very sensitive to UPP inhibition compared to other tested cell lines. As expected, treatment with 4924 caused accumulation of NRF2 (Fig. 3D) in both healthy and FRDA patient-derived lymphoblasts but no effect on FXN – a similar phenotype was observed in 293T cells treated with 4924 (data not shown). We then examined the effect of two compounds, CPD11^7^ and 620301^8^, reported as specific inhibitors of pFXN ubiquitination. Consistent with our inability to increase FXN levels by broad inhibition of the UPP, we were unable to detect any significant effect from treatment with these compounds on p-, i-, or mFXN levels (Fig. S1).

We utilized 293T cells transiently overexpressing FXN to evaluate the role of UPP in FXN turnover under these conditions. Elevated FXN expression clearly influenced the observed phenotypes (Fig. S2). MG132 and BTZ had a very modest effect on iFXN levels in FXN-transfected cells, in contrast to a very robust decrease in untransfected 293T cells (Fig. 3C). Interestingly, UAEi treatment led to very robust stabilization of overexpressed p- and iFXN (Fig. S2), as opposed to loss of iFXN in untransfected cells (Fig. 3C). No effect was observed on GFP protein levels, derived from a co-transfected GFP expression construct, suggesting that compound treatment did not alter vector-derived expression of FXN and GFP (Fig. S2). Our results suggest that excess p- and iFXN are subjected to turnover pathways that are not operative at normal FXN expression levels.

To explore if endogenous FXN is ubiquitinated, we immunoprecipitated FXN from 293T cells treated with BTZ or vehicle (Fig. S3B). A polyubiquitin signal was detected in the anti-FXN immunoprecipitates, which was enhanced by BTZ treatment suggesting that either a subset of FXN or that a co-immunoprecipitated partner is ubiquitinated. To determine if FXN or an interaction partner is ubiquitinated, we repeated endogenous FXN immunoprecipitation and subjected beads to low salt and high salt washes. No poly-ubiquitinated FXN species were detected in anti-FXN immunoprecipitates despite complete immunoprecipitation of FXN protein from lysates derived from DBeQ or BTZ treated cells (Fig. S3C), suggesting that FXN expressed at endogenous levels is not ubiquitinated.

Next, we compared the effect of DBeQ to that of UAEi and BTZ (Fig. 3E). DBeQ caused accumulation of pFXN whereas UAE or proteasome inhibition severely reduced levels of iFXN. Curiously, co-treatment of DBeQ with UAEi or BTZ reversed accumulation of pFXN and diminished levels of iFXN to an almost comparable extent as treatment with UAEi or BTZ alone, suggesting that inhibition of the UPP modulates a FXN regulatory event upstream of p97 (Fig. 3E). Contrary to the effect on FXN, UPP inhibition increased levels of NRF2 and the mitochondrial membrane –associated protein MCL1.

### Modulation of the autophagy pathway does not increase mFXN but alters iFXN levels

**Figure 4:**
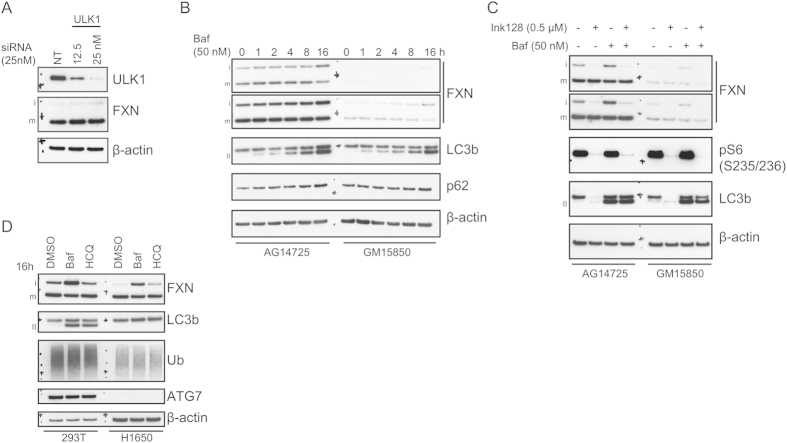
Inhibition of autophagy or autophagic flux does not alter mature frataxin levels. (**A**) ULK1-targeting or non-targeting (NT) control siRNA transfection was carried out in 293T cells at the indicated concentration. Total amount of transfected siRNA was maintained at 25 nM using NT siRNA. 72 h post-transfection, cells were lysed and analyzed by immunoblotting. (**B**) AG14725 or GM15850 lymphoblasts were subjected to a time-course treatment with 50 nM baf. Lysates were prepared and analyzed by western blotting as indicated. Bands corresponding to LC3b-II are denoted with II. (**C**) Lymphoblasts were treated with mTOR inhibitor INK128 (0.5 μM) alone or in combination with baf (2 h post-treatment with INK128). Lysates were analyzed as indicated. (**D**) 293T or H1650 cells were treated with 50 nM baf or 30 μg/ml HCQ for 16 h followed by western blotting. All gels were run under the same experimental conditions, as outlined in the Materials and Methods section.

We used genetic or pharmacological treatments to modulate autophagy at various nodes and examined the impact on FXN levels by western blotting. ULK1 (UNC-51-like kinase 1) is a regulator and a potential early initiator of autophagy^26^. Since knockdown was previously shown to disrupt autophagy^27^,^28^,^29^, we transfected 293T cells with increasing concentrations of ULK1–targeting siRNAs to examine the consequential effect on FXN. At the highest transfected amount, we grossly depleted 293T cells of ULK1 protein (Fig. 4A), but saw no significant effect on mFXN levels. To inhibit autophagy at a later step in the pathway, we used the V-ATPase proton pump inhibitor bafilomycin (baf). Because degradation of autophagosomal contents is a consequence of fusion with lysosomes, inhibitors of lysosomal hydrolysis like baf, also inhibit protein turnover by autophagy. When FRDA lymphoblasts and the corresponding healthy match were treated with baf (Fig. 4B), LC3b-II accumulated as expected, indicative of inhibition of autophagic flux. This was accompanied by accumulation of p62, a known autophagy substrate. mFXN levels did not increase throughout the course of the treatment, however a slight increase in iFXN was observed at the 16 h time point.

To explore the effect of stimulating autophagy on mFXN, we treated control or FRDA patient lymphoblasts with the potent mTORC1/2 inhibitor INK128^30^ (Fig. 4C). These experiments were done with and without baf to investigate if the FXN phenotype was autophagic flux –dependent. mTOR inhibition led to efficient dephosphorylation of the S6 ribosomal protein, indicative of suppression of *de novo* mRNA translation, as well as almost complete loss of LC3b signal due to robust induction of autophagy. Neither INK128 nor the combination with baf yielded any effect on mFXN levels. Interestingly, mTOR inhibition strongly diminished the iFXN signal, similar to treatment with proteasome inhibitors. To determine whether autophagy plays a role in the turnover of iFXN, we utilized an epithelial lung cell line, NCI-H1650 (H1650), with a bi-allelic deletion of the E1 enzyme ATG7, which is essential for both basal and induced autophagy^31^. ATG7 is required for activation of LC3b and other ubiquitin-like substrates in the autophagy pathway^32^. Since H1650 cells do not contain ATG7 protein, they do not accumulate lipidated LC3b upon inhibition of autophagic flux with baf or hydroxychloroquine (HCQ; Fig. 4D) - HCQ is a basic lysosomotropic agent that accumulates in and alters the pH of lysosomes, leading to inhibition of autophagolysosomal fusion and autophagic flux^33^. Inhibition of autophagy by baf or HCQ led to an increase in LC3b-II in 293T cells but not in the autophagy-deficient H1650 cells, consistent with the fact that ATG7 is an essential component of this pathway. However, baf but not HCQ treatment, in the presence or absence of active autophagy, robustly increased iFXN levels. Previous studies had demonstrated that baf treatment influences mitochondrial function^34^, which suggests that the increase in iFXN is likely due to deficient processing of this form to mFXN, and not autophagic impairment.

We considered the possibility that autophagy and proteasomal degradation may play complementary or compensatory roles in protein turnover, as previously reported^35^. To determine if, in the absence of autophagy, proteasome inhibition may alter mFXN levels or that iFXN degradation would be diminished, we subjected H1650 and 293T cells to treatment with an array of proteasomal inhibitors that differentially target 20S proteasome catalysis (Fig. S4). Whereas most of the tested inhibitors modulated levels of proteasomal substrate c-Jun and accumulation of polyubiquitinated proteins in both cell lines, mFXN remained unaltered, further confirming our previous observations that proteasomal degradation does not play a role in regulation of FXN levels. In general, iFXN appears to be reduced following UPP inhibition.

### PITRM1 is a mitochondrial protease that regulates iFXN

**Figure 5:**
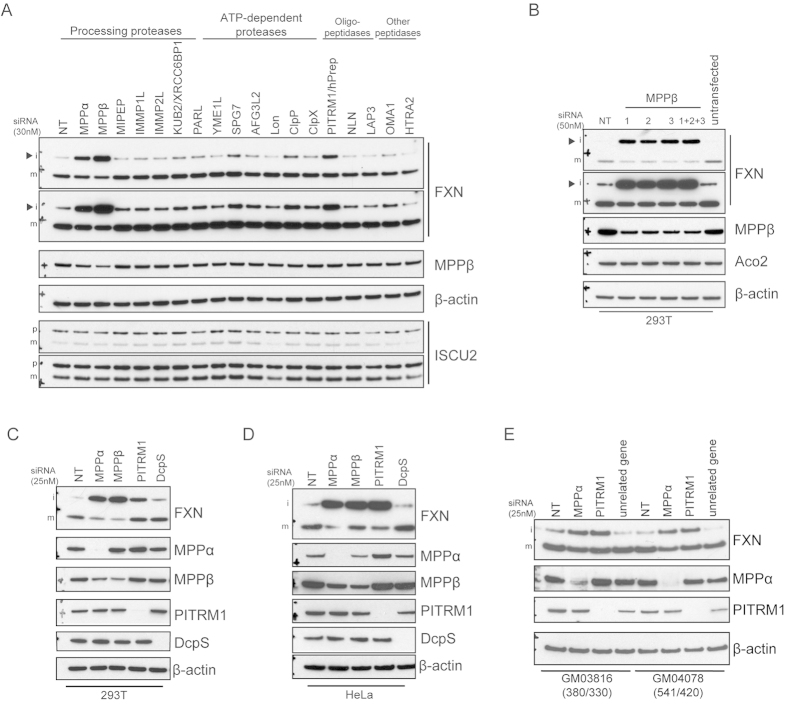
FXN processing and/or proteolysis is regulated by mitochondrial proteases MPPα/MPPβ and PITRM1. (**A**) 293T cells seeded in 12-well plates were transfected with 30 nM siRNA smartpools (Dharmacon) targeting the indicated mitochondrial proteases. 72 h after transfection, cells were harvested and corresponding lysates were analyzed by immunoblotting with the indicated antibodies. Arrowheads mark iFXN. (**B**) Cells were transfected with individual MPPβ-targeting siRNAs or all oligos (1 + 2 + 3) to confirm the effect observed with the MPPβ-targeting smartpool siRNA on FXN processing. Lysates were analyzed by immunoblotting as indicated. (**C**) 293T cells were transfected with 25 nM smartpool siRNA targeting MPPα, MPPβ, PITRM1, with DcpS and NT siRNA as controls. Lysates were analyzed by immunoblotting as indicated. (**D**) HeLa cells were transfected with the same siRNAs as in (**C**) and analyzed with the indicated antibodies. (**E**) FRDA patient-derived fibroblasts GM03816 and GM04078 were transfected with 25 nM siRNA oligos targeting MPPα, PITRM1, an unrelated gene, and an NT control. 96 h later, cells were harvested and lysates were analyzed by immunoblotting. The numbers of GAA expansions in intron 1 of *FXN* (allele 1/allele 2) are noted. All gels were run under the same experimental conditions, as outlined in the Materials and Methods section.

To better understand the complex regulation of FXN, and given the importance of FXN in the Fe-S biogenesis pathway and FRDA, we sought to identify mitochondrial proteases involved in FXN proteolysis. We carried out a limited siRNA screen in 293T cells targeting known mitochondrial proteases and examined levels of FXN and its partner ISCU2 that is also cleaved from a precursor form to yield mature mitochondrial ISCU2 (Fig. 5A). Interestingly, multiple proteases regulated levels of iFXN. As expected, knockdown of MPPα and MPPβ, both of which are implicated in processing of iFXN to its mature form, inhibited maturation of FXN. Partial knockdown of MPPβ (~50%) strongly inhibited iFXN processing. However, no accumulation of pFXN was observed despite reported evidence for the involvement of MPP in pFXN processing^13^. Reduction in the levels of a novel FXN-regulating protease, PITRM1, showed significant modulation of iFXN levels. PITRM1 is thought to degrade unstructured peptides and has been implicated in the cleavage of amyloid beta (Aβ) on accumulation in mitochondria^36^. Two others, SPG7/paraplegin and ClpP showed modest effects but were not further pursued. Lon knockdown caused diminished levels of mature ISCU2 but had no effect on FXN.

We performed additional experiments to confirm the effects of MPPα, MPPβ, and PITRM1 knockdown. Individual siRNA oligos targeting MPPβ modulated iFXN processing (Fig. 5B). Similar to the siRNA mixed pool transfections, robust accumulation of iFXN was observed yielding a dramatic increase in total FXN amounts (mFXN and iFXN combined). We also carried out siRNA transfections with four individual oligos targeting PITRM1 (Fig. S5A) or MPPα (Fig. S5B). Successful knockdown of PITRM1 or MPPα with these oligos led to an increase in iFXN levels, similar to mixed pool transfections. Moreover, knockdown of MPPα, MPPβ, or PITRM1 yielded similar effects in multiple cell lines (Fig. 5C,D), in addition to FRDA fibroblasts (Fig. 5E), suggesting a global role for these proteases in regulation of FXN levels.

## Discussion

Maintenance of sufficient intracellular FXN protein is physiologically critical for constant Fe-S cluster synthesis and for Fe-S enzyme catalysis^4^. Little is known about the role of protein degradation in regulating FXN levels. The presence of three forms of FXN, pFXN, iFXN, and mFXN in cellular compartments accessible to different proteases further complicates delineation of the degradative pathways that control corresponding levels. In an initial attempt to understand pharmacological modulation of the different forms of FXN and total levels, we investigated if p97/VCP, the UPP and/or autophagy are involved in FXN turnover. That cells can process and accumulate excess mFXN compared to control endogenous levels suggested that the maturation machinery is not rate limiting. We treated multiple cell types, including FRDA patient-derived cells, with small molecules that target important nodes in these pathways. Among the many conditions tested, none increased mFXN levels (Table 1). In contrast, our data clearly show that levels of pFXN and iFXN can be altered by proteostasis modulators. Taken together, these results suggest that p97, the UPP, or autophagy do not regulate mFXN levels. Importantly, our central conclusion is that such chemical approaches can alter total FXN levels but mFXN is surprisingly difficult to modulate.

The effects produced by modulating proteostasis are complex. p97 has been implicated in pleiotropic cellular functions, including key roles in proteostasis^16^. Targeting p97 with the inhibitor DBeQ produced robust accumulation of pFXN. A similar phenotype was observed by mitochondrial membrane uncoupling. These data suggest the possibility that modulation of p97 or mitochondrial membrane potential may influence mitochondrial import of pFXN, because accumulation of pFXN influenced iFXN levels. Steady state levels of iFXN were modulated by several of the treatments; however, the results are difficult to explain mechanistically. Inhibition of the proteasome or mTOR strongly diminished levels of iFXN in 293T cells. Conversely, treatment with baf increases iFXN levels. Further characterization of the mechanisms that govern iFXN levels would be very interesting, as this may serve as an important checkpoint and an important determinant of endpoint mFXN levels. A cryptic turnover step was previously reported for mutant iFXN W173G^13^. In 293T cells, treatment with the translation inhibitor cycloheximide (CHX) caused depletion of iFXN without an observable effect on mFXN and pFXN (Fig. S3A), suggesting that iFXN may at least partially be turned over by a specific degradative mechanism. Whether such an iFXN turnover pathway exists remains to be better elucidated. Curiously, we observed a reduction of mFXN in BTZ -treated lymphoblasts (Fig. 3D) which is surprising given the long half-life of mFXN. While this indicates that there is a UPP-independent pathway of mFXN turnover in lymphoblasts, the significance is hard to assess, as BTZ treatment did not reduce mFXN in other cell types.

Proteostasis modulators gave different patterns of p-, i-, and mFXN levels in cells overexpressing FXN compared to cells expressing endogenous levels. We showed that UAE inhibition leads to the expected increase in NRF2 and c-Jun, both substrates of the UPP, however no increase in any of the FXN forms was observed when examining endogenously expressed protein. Furthermore, no ubiquitinated FXN could be detected in FXN immunoprecipitates. This contradicts the effect of the same treatment on cells transiently overexpressing FXN, suggesting that proteasomal degradation may actively remove surplus FXN without necessarily contributing to endogenously expressed FXN turnover. Indeed, a recent study identified proteasomal engagement to clear excess precursor mitochondrial proteins in yeast as an extension of the unfolded protein response to cope with proteostatic stress^37^. Care must therefore be taken when evaluating involvement of such pathways in modulation of protein levels, especially when potential substrates are overexpressed. Additionally, our data suggest that endogenous FXN, in contrast to overexpressed FXN, cannot be stabilized through inhibition of critical nodes in this pathway. Therefore, from a drug discovery perspective, it is unclear that targeting this pathway will be of utility for FXN upregulation.

In the absence of a clear role for the UPP and/or autophagy in mFXN regulation, we explored the possibility that one or more mitochondrial proteases may regulate this process. To further understand FXN proteolysis, we performed a genetic screen targeting known mitochondrial proteases and identified MPPα, MPPβ, and PITRM1 as modulators of FXN levels. A role for MPPβ has been previously described in the processing of pFXN and iFXN to yield mFXN. MPPα is a component of the MPP heterodimer complex that is involved in substrate recognition^38^, so diminished levels were expected to yield an effect on FXN processing. In our experiments, no accumulation of pFXN was observed following MPPα or MPPβ knockdown suggesting that, in the cellular context, other factors may be required to catalyze pFXN processing, or that the knockdown efficiency of MPPβ (~50%) was not sufficient to yield that effect. Interestingly, a mutation was recently identified in *PMPCA*, the gene that encodes MPPα, in patients suffering from a non-progressive form of ataxia^39^. Mutant *PMPCA* patient-derived cells accumulate significant iFXN such that total FXN exceeds the level in healthy cells^39^, consistent with data from MPPα knockdown experiments reported here. We also identified PITRM1 as a putative novel FXN protease. That PITRM1 activity can be inhibited by oxidizing agents^40^ suggests a possibility that it may be prone to modulation under oxidative stress, such as in FRDA^41^. Additionally, Aβ was recently shown to inhibit the yeast orthologue of PITRM1 leading to dysfunctional protein processing^42^. Whether PITRM1 directly or indirectly regulates iFXN, or is involved in the processing of iFXN to mFXN remains to be determined. Interestingly, diminished expression of any of the three enzymes yielded increases in iFXN levels, the outcome of which is an increase in total FXN levels. Together, the data further suggests the presence of a potential degradative checkpoint at the iFXN level, in addition to processing to mFXN. Because both PITRM1 and MPPβ are metallopeptidases, we investigated if treatment with iron chelators, which previously were suggested to have therapeutic utility in FRDA^43^, would yield a similar phenotype. Treatment with the chelating agent deferoxamine (DFO) led to an increase in iFXN levels similar to that observed in our MPPβ knockdown experiment (data not shown), suggesting modulation of FXN processing. It remains uncertain whether treatment with chelators is beneficial for FRDA^44^,^45^.

Our data indicate that processing of FXN is a sensitive process which can be modulated by chemical and genetic perturbations of proteostasis. Changes in total FXN are driven by changes in the pFXN and iFXN forms whose functions and abundance in tissues is unclear. It is important to note that commercial FXN quantification kits measure total FXN levels using antibodies that target epitopes in mFXN, which are shared with pFXN and iFXN^46^,^47^. These are useful to quantify FXN levels among patients. However, in the context of FRDA drug discovery, an increase in total FXN levels induced by small molecule treatment does not necessarily imply elevated levels of mFXN. While a substantial body of evidence shows that mFXN is functional, it remains unclear whether iFXN is simply a processing intermediate or whether it shares functions with mFXN. In yeast, a mutant of the yeast FXN orthologue YFH1 that is not processed to the mature form was reported to rescue viability of *∆yfh1* yeast, as well as the defective iron import phenotype associated with loss of *yfh1*^48^, suggesting iFXN is active and that it has functional redundancy with mFXN. The results of experiments testing the role of human iFXN are more difficult to interpret. Human iFXN and mFXN can both interact *in vitro* with components of the Fe-S cluster synthesis machinery^19^; however, in cells, a processing mutant showed limited rescue of a FXN knockout^49^. In patients carrying a *PMPCA* mutation that destabilizes MPPα, mFXN levels are reduced to levels similar to some FRDA patients^39^. Interestingly, cells from the former patients accumulate iFXN and patients display a non-progressive form of ataxia, two features that distinguish it from FRDA^39^. A possible explanation for the milder ataxia phenotype is that iFXN shares some of the functions of mFXN, and thus the higher levels of iFXN compensate for mFXN deficiency. Whether iFXN has unique functions is unknown. Altogether, our data show that levels of p- and iFXN can be pharmacologically modulated; therefore we suggest that chemical agents that increase FXN be characterized for their effects on all forms of FXN.

## Materials and Methods

### Chemicals

BTZ, DBeQ, and INK128 were obtained from Selleckchem; MLN4924 from Cayman Chemicals; Epoxomicin, Ac-Ala-Pro-Nle-Asp-al, Clasto-Lactacystin beta-lactone, Ada-(Ahx)3-Leu3-vinylsulfone, and MG-132 from Enzo; CCCP, CHX, baf, and HCQ from Sigma. UAEi was synthesized as detailed previously^50^.

### Cell culture

293T, HeLa, and H1650/NCI-H1650 cells were purchased from ATCC (American Type Culture Collection) and grown in RPMI1640 (Invitrogen) supplemented with 10% Fetal Bovine Serum (FBS; Invitrogen Cat# 16000-044) and 20 mM HEPES buffer (Invitrogen). Patient-derived or control lymphoblasts GM15849, GM15850, and AG14725 were obtained from the NIGMS Human Genetic Cell Repository at the Coriell Institute for Medical Research and grown in RPMI1640 supplemented with 10% FBS and 20 mM HEPES. Patient-derived or control fibroblasts GM03816, GM04078, and AG09309 were also obtained from the Coriell Institute for Medical Research and grown in DMEM (Invitrogen) supplemented with 10% FBS and 20 mM HEPES.

### Cell lysate preparation and western blotting

For adherent cells, including 293T, fibroblasts, HeLa, and H1650, plates were washed with ample PBS and then freshly made lysis buffer (0.5% Igepal CA-630 from Sigma, 50 mM TrisCl pH 7.5, 150 mM NaCl) supplemented with protease cocktail cOmplete mini EDTA-free tablets (Roche), phosphatase inhibitor cocktail PhosSTOP (Roche) and 10 mM of the Cys alkylating agent N-ethylmaleimide (NEM, Thermo) was applied. Cells were scraped with a cell lifter (Corning) where required and transferred to a clean microtube. Lysates were vortexed, left on ice for 30 mins, and then subjected to freeze thaw lysis before clearing by centrifugation at 21,000 × g for 10 mins at 4 °C. For suspension-grown lymphoblasts, cells were centrifuged at 500 × g to remove cell culture media then washed in PBS. Pellets were resuspended in the lysis buffer described above and subjected to the same lysis conditions. Protein concentrations of the cleared cell lysate supernatants (sups) were then determined using the protein 660 nm protein assay reagent (Pierce) compared to bovine serum albumin standards (Pre-diluted protein assay standard BSA set, Pierce).

### Plasmids, siRNA and transfections

Full-length human FXN cDNA sequence was synthesized (Genewiz) and subcloned into pCDNA3.1 Hygro (Invitrogen). For 293T transfections, cells were seeded at 500,000 cells/well in 6-well plates overnight and then transfected using Fugene 6 (Promega; 3 μl/well) and 1 μg total DNA per well. For fibroblasts, cells were seeded at 200,000 cells/well in 6-well plates and plasmid reverse transfections were carried out immediately using TransIT X2 (Mirus; 5 μl/well) and 2.5 μg total DNA per well. Cells were harvested as noted at the indicated post-transfection time points. siRNA oligo smartpools (Dharmacon) and individual oligos (source and target sequences listed in Supplementary Table 1) were transfected into 293T cells or patient fibroblasts seeded in 6-well plates using Dharmafect I (Dharmacon; 5 μl/well) and the indicated concentration of oligo. Cells were later harvested at the indicated post-transfection time points.

### Antibodies and western blotting

All antibodies utilized for western blotting in this report are listed in Supplementary Table 2 with corresponding source, catalog number, and dilution. For immunoblotting, typically 5–10 μg of total lysate were loaded per lane and resolved on a 4–12% NuPAGE BisTris gel (Invitrogen) with MES SDS running buffer (Invitrogen) supplemented with NuPAGE anti-oxidant. Proteins were transferred to a 0.2 μm nitrocellulose membranes (Bio-Rad) using the Transblot Turbo system (Bio-Rad). Membrane was then incubated in blocking buffer (5% non-fat dry milk from Cell Signaling reconstituted in TBS supplemented with 0.05% Tween20) for 1 h at room temperature. Primary antibodies were diluted in blocking buffer at the concentrations indicated in Supplementary Table 2 and added to membrane overnight at 4 °C. Next day, membranes were washed three times with ample TBST (TBS supplemented with 0.05% Tween20) and incubated in secondary antibody diluted in blocking buffer for 30 min at room temperature. HRP-conjugated anti-Rabbit IgG antibody (Invitrogen 656120) and anti-Mouse IgG antibody (Invitrogen G21040) were diluted in blocking buffer at 1:7500 and 1:5000, respectively. Membranes were finally washed four times in ample TBST and signal was developed using the SuperSignal West Pico or Femto (Pierce), or ECL Prime (GE) chemiluminescent substrates before exposure to film (Biomax, Kodak).

### FXN immunoprecipitation

Anti-FXN antibody ab110328 (abcam) was utilized for immunoprecipitation of FXN protein from lysates. To cross-link antibodies to protein A/G beads (Santa Cruz Biotech), 500 μl resin was washed in PBS and then incubated with 100 μl ab110328 antibody for 1 h at room temperature with end-over-end rotation. Beads were then washed with PBS and covalent conjugation of antibody was carried out using a freshly prepared primary amine cross-linking buffer (180 μl 2.5 mM DSS from Pierce, 770 μl water, and 50 μl 20 × PBS). Residual DSS and unbound antibody were removed by washing with 0.1 M Glycine pH 2.5–3 followed by washes in PBS. Finally, beads were stored at 4 °C in PBS. For immunoprecipitation (IP), 500 μg of cleared cell lysates (containing NEM) were incubated with 25 μl of anti-FXN conjugated beads for 2 h at 4 °C then washed two times with lysis buffer (Fig. S3B) or in high (500 mM NaCl, 50 mM TrisCl pH 7.5, 0.5% Igepal CA-630) and low salt buffers (10 mM TrisCl, 0.5% Igepal CA-630; Fig. S3C) before removal of trace amounts of lysis buffer. Resultant IP beads were resuspended in 50 μl 2x LDS sample buffer and heated at 100 °C for 5 min. Eluates were then carefully transferred, without beads, to clean microtubes and supplemented with DTT reducing agent. Input lysates, IPs, and output flowthrough lysates were analyzed by immunoblotting as described above.

### Resolution of (±) Compound11

Racemic Compound 11 (CPD11)^7^ obtained commercially (TimTec ST083644) was separated into two enantiomers by SFC (supercritical fluid chromatography) with methanol, acetonitrile or isopropyl alcohol/hexane mixture as eluants. An immediate inter-conversion of enantiomers (~20% conversion) was observed in liquid media after the separation, and a full racemization occurred approximately 1 h after separation. Racemization was observed in all solvents with a similar epimerization rate. A chiral separation was attempted by forming tartrate salts of CPD11 using a standard separation protocol, but the optical rotation values of the collected solids suggested a racemic material. CPD11 was also found to readily decompose in methanol and other organic solvents at elevated temperatures (>50 °C).

## Additional Information

**How to cite this article**: Nabhan, J. F. *et al.* Perturbation of cellular proteostasis networks identifies pathways that modulate precursor and intermediate but not mature levels of frataxin. *Sci. Rep.* **5**, 18251; doi: 10.1038/srep18251 (2015).

## Supplementary Material

Supplementary Information

## Figures and Tables

**Table 1:** Effects of various chemical treatments on FXN levels.

Inhibitor	Target	pFXN	iFXN	mFXN	p+i+m	Note
DBeQ	p97/VCP	+++	−	No effect	+	
CCCP	Mitochondrial membrane	+++	−	No effect	+	
UAEi	UAE/E1	No effect	Variable	No effect	Variable	− − − iFXN in 293T, no effect in lymphoblasts
MLN4924	NAE	No effect	No effect	No effect	No effect	
BTZ/MG132	20S proteasome	+	− − −	Variable	Variable	− mFXN in lymphoblasts, no effect in 293T
Bafilomycin	V-ATPase	No effect	+	−	No effect	
HCQ	lysosome	No effect	No effect	No effect	No effect	
INK128	mTORC1/2	No effect	− − −	No effect	− −	
DFO	Fe chelation	No effect	+++	No effect	++	

Inhibitors and targets are listed along with the corresponding effect of treatment on various forms of FXN (pFXN, iFXN, and mFXN) and total FXN levels. +, ++, +++ denote a modest, significant, or robust increase in levels of a specific form of FXN, respectively. −, − −, − − − denote a modest, significant, or robust decrease in levels of a specific form of FXN, respectively.
