# Supplementary material for: Perturbation of cellular proteostasis networks identifies pathways that modulate precursor and intermediate but not mature levels of frataxin
Source: Sci Rep. 2015 Dec 16;5:18251. doi: 10.1038/srep18251 (PMC4680912; doi:10.1038/srep18251)
Supplement: Supplementary Information [file srep18251-s1.pdf]

Perturbation of cellular proteostasis networks identifies pathways that modulate precursor and intermediate but not mature levels of frataxin

Joseph F Nabhan*, Renea L Gooch, Eugene L Piatnitski Chekler, Betsy Pierce, Christine E Bulawa

*corresponding author: joseph.nabhan@pfizer.com

SUPPLEMENTARY INFORMATION

Supplementary Figures

Figure S1. Putative frataxin ubiquitination inhibitors/competitors yield no effect on endogenous mature frataxin protein enrichment. We evaluated if a more targeted approach can modulate FXN levels. Two previous reports suggested that pFXN is a UPP substrate and utilized computational screening approaches to identify putative ubiquitination inhibitors of FXN [1,2]. A) We tested the effect of putative FXN ubiquitination inhibitor CPD11 [1] on endogenous mFXN levels. A dose-response study using the racemic mixture of CPD11 in 293T cells failed to yield any significant effect on precursor, intermediate or mature FXN levels. B) We then explored the potential ability of a combination approach of CPD11 with UAEi or BTZ to enrich cells with mFXN. We reasoned that this combination treatment may potentiate a stabilization of pFXN effect. Cells were subjected to a 24 h treatment with CPD11 and supplemented with UAEi or BTZ during the last 6 h or incubation. Western analysis of the lysates demonstrated robust accumulation or loss of polyubiquitinated proteins upon treatment with BTZ or UAEi, respectively. However, consistent with previous experiments using BTZ or UAEi (Figure 3), we only observed a decrease in iFXN but no change in endogenous mFXN levels. C) We attempted to isolate the putative active CPD11 enantiomer but were unsuccessful due to rapid decomposition in less than 10 min at both 20°C and 37°C at physiological or near physiological pH. D) We also tested a representative molecule, 620301, from another class of putative FXN ubiquitination inhibitor molecules [2] for its ability to increase endogenous mFXN. Lymphoblasts heterozygous for the GAA expansion in intron 1 of FXN (GM15849) or homozygous for the expansions (GM15850) and a healthy match (AG14725) were subjected to a dose-response treatment with 620301 for 5 days. Similar to the results for CPD11, no changes to pFXN, iFXN or mFXN levels were observed by western analysis in any of the lymphoblast lines.

Figure S2. Inhibition of the UPP alters overexpressed precursor and intermediate but not mature FXN protein levels. To test the effect of UPP inhibition on overexpressed FXN, 293T cells were seeded in 6-well plates and co-transfected with 0.2 µg hFXN plasmid and 0.1 µg GFP expression construct per well for 24 h (supplemented to 1 µg total plasmid per well with EV) before being subjected to a 16 h treatment with the indicated final concentrations of UAEi, BTZ, MG132, or DMSO vehicle. Cells were harvested and analyzed by immunoblotting.

Figure S3. Precursor frataxin can be modestly increased by proteasome inhibitors but no ubiquitinated FXN could be detected in our system. A) 293T cells were treated for 24 h with BTZ or MG132 (10 μM) and corresponding lysates were analyzed by western blotting. All three forms of FXN (precursor, intermediate, and mature) are detected. B) Lysates from 293T cells treated with 10 μM BTZ for 24 h were subjected to an anti-FXN immunoprecipitation reaction using anti-FXN (abcam, ab110328) DSS cross-linked beads. Input lysates (Input), immunoprecipitates (IP) and flowthrough (FT) were analyzed by western blotting as indicated. C) 293T cells treated with 10 μM DBeQ, 10 μM BTZ, or 0.1% DMSO vehicle were harvested in NEM-containing lysis buffer, as detailed in methods, and subjected to immunoprecipitation with anti-FXN conjugated beads as in B). Beads were washed with high salt (500 mM NaCl, 50 mM Tris-HCl pH 7.5) buffer and low salt (10 mM TrisCl pH 7.5) buffers. Input lysates (Input), immunoprecipitates (IP), and flowthrough lysates (FT) were analyzed by immunoblotting as indicated.

Figure S4. Inhibition of the proteasome in the presence or absence of functional autophagic protein turnover does not alter mature frataxin protein levels and reduces intermediate frataxin levels. H1650 is a macroautophagy-defective ATG7-null cell line. 293T or H1650 cells were treated with the indicated proteasomal inhibitors (1 μM; Vinyl Sulf.: Ada-(Ahx)3-Leu3-vinylsulfone, Lactacys.: Clasto-Lactacystin beta-lactone, Epox.: Epoxomicin, MG132, Ac-A-P. :Ac-Ala-Pro-Nle-Asp-al) for 24 h and then analyzed by immunoblotting as indicated.

Figure S5. Knockdown with individual siRNA oligos targeting MPPα or PITRM1 leads to modulation of intermediate FXN levels. 293T cells were transfected with individual oligos (25 nM) targeting A) PITRM1 or B) MPPα. In both instances, successful knockdown of the target gene led to an increase in levels of iFXN. Arrowheads indicate iFXN immunopositive bands.

Supplementary Tables

Supplementary Table 1. List of individual siRNA oligo sequences.

Supplementary Table 2. List of utilized antibodies and corresponding dilutions for western blot analysis.

References (Supplementary material)

1 Lavecchia, A. *et al.* Discovery of a novel small molecule inhibitor targeting the frataxin/ubiquitin interaction via structure-based virtual screening and bioassays. *Journal of medicinal chemistry* **56**, 2861-2873, doi:10.1021/jm3017199 (2013).

2 Rufini, A. *et al.* Preventing the ubiquitin-proteasome-dependent degradation of frataxin, the protein defective in Friedreich's ataxia. *Human molecular genetics* **20**, 1253-1261, doi:10.1093/hmg/ddq566 (2011).

Figure S1. Putative Frataxin ubiquitination inhibitors/competitors yield no effect on endogenous mature Frataxin protein enrichment.

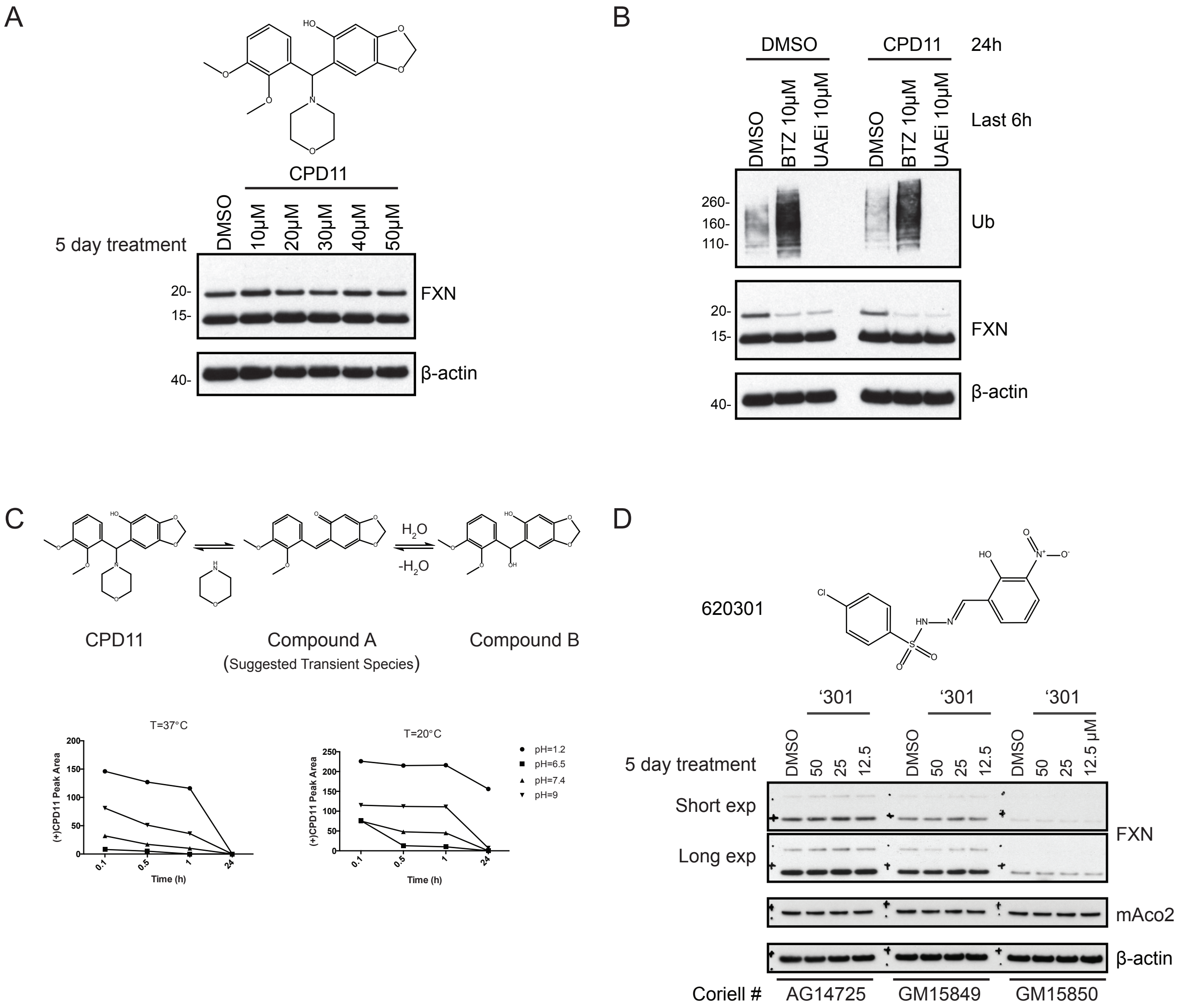

Figure S2. Inhibition of the UPP alters overexpressed precursor and intermediate but not mature FXN protein levels.

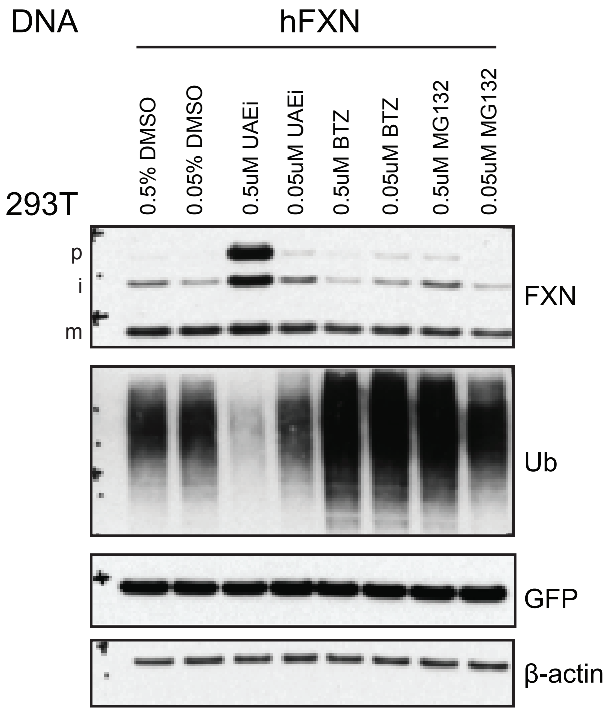

Figure S3. Precursor Frataxin can be modestly increased by proteasome inhibitors but no ubiquitinated FXN could be detected in our system.

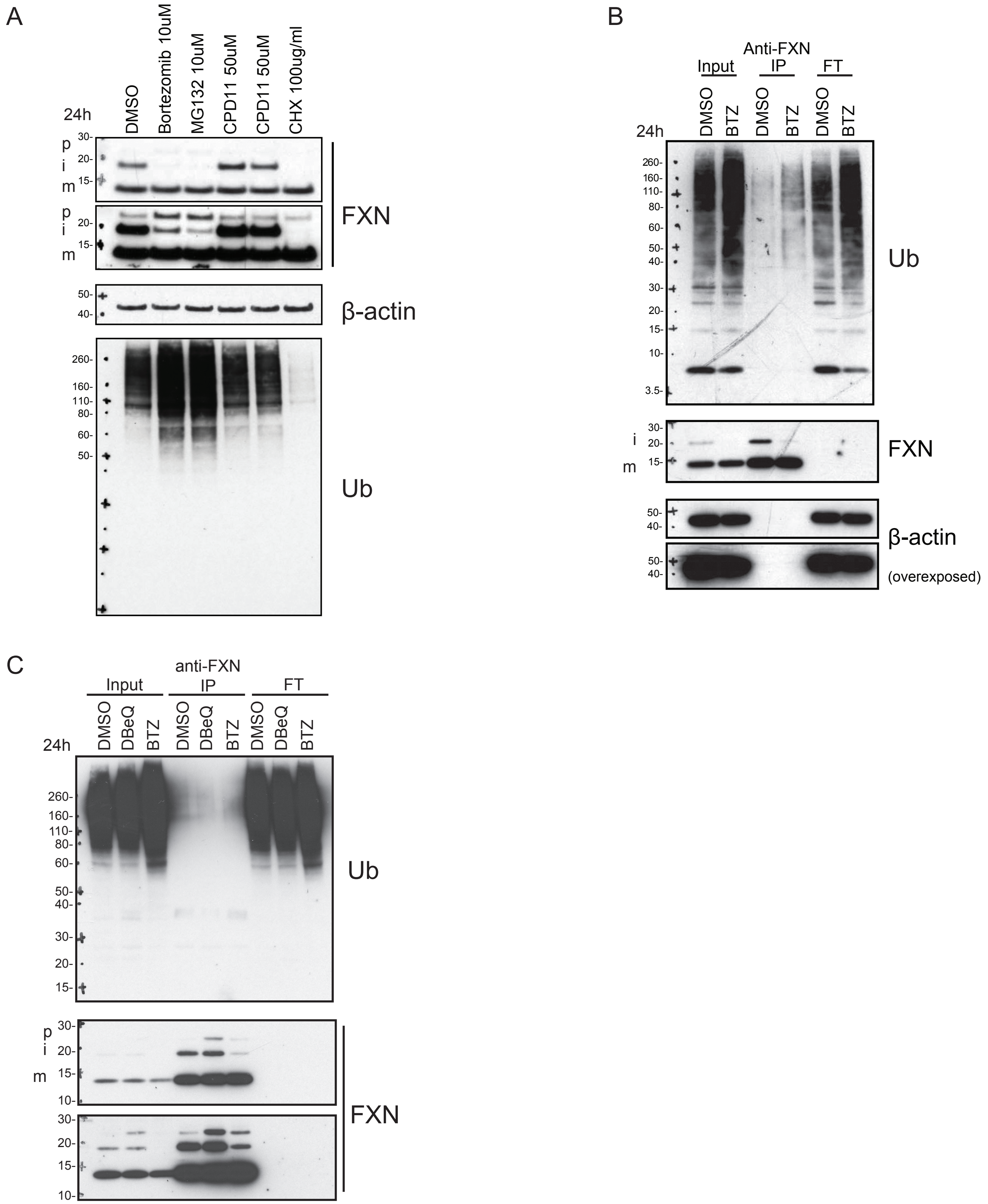

Figure S4. Inhibition of the proteasome in the presence or absence of functional autophagic protein turnover does not alter mature Frataxin protein levels and reduces intermediate Frataxin levels.

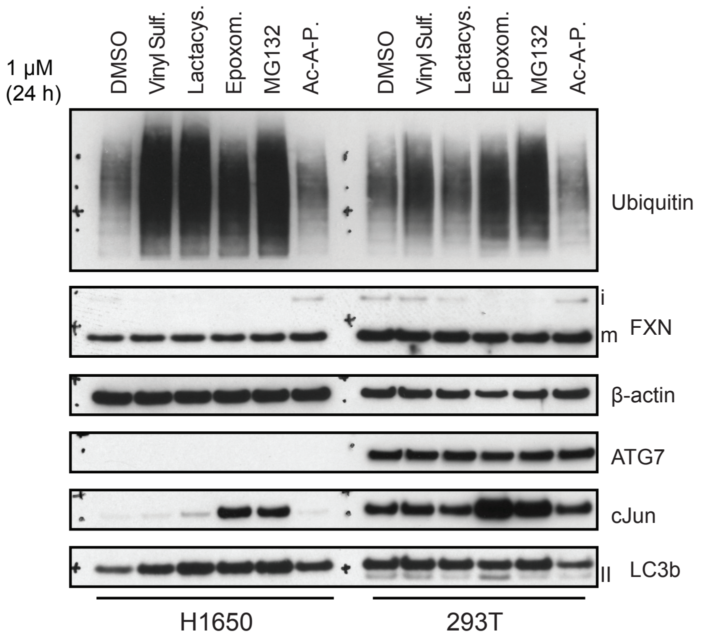

Figure S5. Knockdown with individual siRNA oligos targeting MPPα or PITRM1 leads to modulation of intermediate FXN levels.

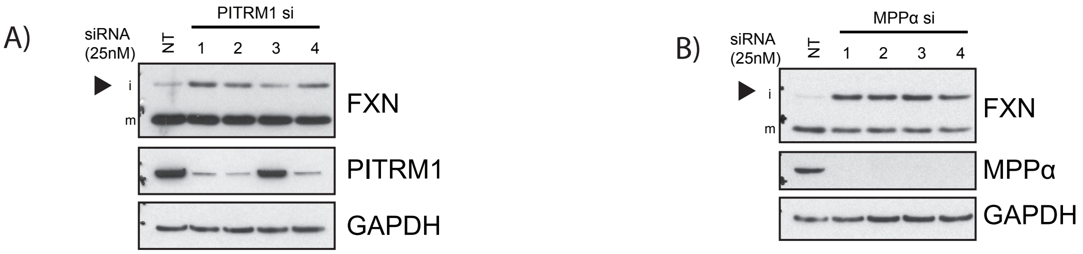

Supplementary Table 1. List of individual siRNA oligo sequences.

Target name	Oligo target sequences	Source
p97/VCP	1: GCAUGUGGGUGCUGACUUA<br>2: CAAAUUGGCUGGUGAGUCU	Dharmacon
ULK1	proprietary (Cat# 7000)	Cell Signaling
MPPα	1: CGAGAUAUGAAGCGAAAUA<br>2: AGACCGCCCUGUCGAGUAA<br>3: GCGCAGAGGCCGUGGAUAU<br>4: GGACACGGUGGUUGCCUUA	Dharmacon
MPPβ	1: GCAAGAAUUGAUGCUGUGA<br>2: GACUUAGUGGAUUAUAUAA<br>3: CAGAUACGCAGUAACAUGU	Sigma
PITRM1	1: CGAGAAAGGUCUAGAAUUA<br>2: GAAGUUGGGAAAUCAGUUA<br>3: CAGAACGAUUGAUGAAGUA<br>4: CCUAGGGAAUUCCAGAUAA	Dharmacon

Supplementary Table 2. List of utilized antibodies and corresponding dilutions for western blot analysis.

Protein name	Observed MW (KDa)	Source	Cat #	Ab dilution
Aco2	~80	abcam	ab129069	1:12,000
ATG7	~72	abcam	ab52472	1:12,000
c-Jun	40	abcam	ab32137	1:1000
DcpS	40	abcam	ab57314	1:1000
FXN	14-21	abcam	ab110328	1:1000
GAPDH	38	Cell Signaling	8884S	1:12,000
GFP	~28	Cell Signaling	2956S	1:2000
ISCU2	15-20	Proteintech	14812-1-AP	1:6000
LC3b	~15	Sigma	L7543	1:3000
MCL1	~40	Cell Signaling	5453	1:2000
MPPα	~55	abcam	ab14171	1:2000
MPPβ	~50	Proteintech	16064-1-AP	1:2000
NFS1	~50	Proteintech	15370-1-AP	1:2000
NRF2	~90	abcam	ab62352	1:1000
p62	~58	BD Biosciences	610832	1:1000
p97	~100	Cell Signaling	2649S	1:1000
PITRM1	~110	abcam	ab111681	1:2000
pS6 (S235/236)	30	Cell Signaling	4858S	1:1000
Tim23	~22	Proteintech	11123-1-AP	1:1000
Ubiquitin	high mw smear	Cell Signaling	3936S	1:1000
Ubiquitin K48 chains*	high mw smear	Millipore	05-1307	1:12,000
ULK1	~140KDa	Cell Signaling	6439S	1:1000
β-actin	42	Cell Signaling	5125S	1:12,000

* anti-UbK48 was used to probe effect of UAEi, BTZ, or MLN4924 on global levels of protein ubiquitination.
